# Retraction: Differential SELEX in Human Glioma Cell Lines

**DOI:** 10.1371/journal.pone.0228838

**Published:** 2020-02-12

**Authors:** The PLOS ONE Editors

Following the publication of this article [1], concerns have been raised regarding Figure 5:

- In Figure 5A, the upper left pcyclin D1 panel, the band in lane 5 appears similar to the band in lane 6, if stretched horizontally.
- In Figure 5A, the upper left cyclin D1 panel, the band in lane 5 appears similar to the band in lane 6, if stretched horizontally.
- In Figure 5A, the lower left pcyclin D1 panel, the bands in lane 1, 2, 3, 5, and 6 appear similar.
- In Figure 5A, the lower left cyclin D1 panel, the band in lane 1 appears similar to the band in lane 6 when horizontally flipped.
- In Figure 5A, the upper right pcyclin D1 panel, the bands in lane 1, 2, 4, and 5 appear similar.
- In Figure 5B, the upper left ERK panel appears similar to the lower left ERK panel if the lower panel is stretched horizontally.
- In Figure 5B, the lower left pERK panel, the bands in lane 1, 2, 5, and 6 appear similar.
- In Figure 5B, the upper right pERK panel, the bands in lane 1, 2, 4, and 5 appear similar.
- In Figure 5B, the upper right ERK panel, the bands in lane 1, 2, and 4 appear similar.
- Multiple vertical discontinuities in the background of the blots have been detected in the following panels:
  - Figure 5A, pcyclin D1 upper left, lower left, and upper right panels.
  - Figure 5A, cyclin D1 upper left, lower left, and upper right panels.
  - Figure 5B, pERK upper left, lower left, and upper right panels.
  - Figure 5B ERK upper right panel.

Original data for the western blots presented in the paper are no longer available. Replication data have been provided in support of results in Figure 5A, pcyclin D1/cyclin D1/αtubulin upper left, lower left, and upper right panels as well as Figure 5B pERK/ERK upper right and lower left panels. However, these data did not resolve the concerns about western blot data reporting in the original figure.

In light of the unresolved concern about Figure 5 that calls into question the integrity of the reported results, the *PLOS ONE* Editors retract this article.

AHA, BT did not respond. LC, CLE, and VdF disagree with retraction and maintain that all the findings of the work reported in this article are still reliable.

## References

[1] Cerchia L, Esposito CL, Jacobs AH, Tavitian B, de Franciscis V (2009) Differential SELEX in Human Glioma Cell Lines. PLoS ONE 4(11): e7971 10.1371/journal.pone.0007971 19956692 PMC2776989

